# Comorbidities and polypharmacy among HIV-positive patients aged 50 years and over: a case–control study

**DOI:** 10.1186/s13104-019-4576-6

**Published:** 2019-09-03

**Authors:** José Antonio Mata-Marín, Moisés Hermilo Martínez-Osio, Carla I. Arroyo-Anduiza, María de los Ángeles Berrospe-Silva, Alberto Chaparro-Sánchez, Itzel Cruz-Grajales, Javier Enrique Cruz-Herrera, Luis Antonio Uribe-Noguez, Jesus E. Gaytán-Martínez, Medardo Jerónimo-Morales

**Affiliations:** 1 Infectious Diseases Department, Instituto Mexicano del Seguro Social, Hospital de Infectología, “La Raza” National Medical Center, IMSS, Seris y Jacarandas s/n, colonia la raza, Del Azcapotzalco, PO. 02990 Mexico City, Mexico; 2 Internal Medicine Department, Hospital General, “La Raza” National Medical Center, IMSS, Mexico City, Mexico; 3 Infectious Diseases Department, Instituto Mexicano del Seguro Social, Unidad de Infectología “Juan I. Menchaca”, Ext HGR 45, IMSS, Guadalajara, Jalisco Mexico; 4 Clinical Pathology Department, Banco Central de Sangre, Hospital de Especialidades, “La Raza” National Medical Center, IMSS, Mexico City, Mexico; 5 Internal Medicine Department, Hospital HGZ-# 3, Querétaro, Mexico

**Keywords:** Comorbidity, HIV, Polypharmacy

## Abstract

**Objective:**

This study was to determine and compare the prevalences of polypharmacy and comorbidities in patients aged 50 years or older with those patients younger than 50 years in a Mexican population.

**Results:**

One hundred and twenty-five patients were enrolled, 60 (48%) were aged 50 years or older. The median CD4+ cell counts were 509 cells/μL (interquartile range [IQR]: 324–730) for the older patients and 384 cells/μL (IQR: 262–562) (*P* = 0.021) for the younger patients. Viral suppression were significantly higher in the older group: 80% vs. 63% (*P* = 0.037). The number of comorbidities was significantly higher in the older group, with a median of 2 (IQR: 2–3) vs. 1 (IQR: 0–1) (*P* ≤ 0.001). After adjustment of the logistic regression model in the older group, the following comorbidities differed between the age groups: systemic arterial hypertension (odds ratio [OR]: 15.75; 95% confidence interval [CI] 3.49–71.05; *P* = < 0.001), diabetes mellitus (OR: 14.36; 95% CI 1.79–115.07; *P* = 0.001), osteoarthritis (OR: 10.33; 95% CI 2.88–37.05; *P* = < 0.001), hyperlipidemia (OR: 2.78; 95% CI 1.22–6.34; *P* = 0.001), and polypharmacy (OR: 6.58; 95% CI 3.01–14.39; *P* = 0.001).

## Introduction

The survival rate and number of people infected with human immunodeficiency virus 1 (HIV-1) who are older than 50 years have increased since the introduction of antiretroviral therapy (ART) in the 1990s [1]. An unexpected increase in the number of comorbidities, such as diabetes mellitus, systemic arterial hypertension, obesity, and dyslipidemia, which are associated with aging, has been documented in older people with HIV-1. Consequently, the number of drugs used and prevalence of polypharmacy among older HIV-infected people have also increased [2].

The number of comorbidities, level of polypharmacy including ART, and number of physiological age-related changes in various organs and systems can lead to adverse effects and drug interactions, which may affect the clinical condition, lifespan, and quality of life of older HIV-infected people [3]. Several reports have suggested that ART and HIV-1 may increase the risk of chronic comorbid diseases such as hyperlipidemia, atherosclerosis, and osteoporosis [4, 5]. One study found that HIV-infected patients older than 55 years had, on average, four more comorbidities than patients who were younger than 45 years [6].

Many comorbidities are associated with increased drug use, and polypharmacy is associated with an increased risk of mortality in elderly patients [7]. This risk may be higher in people infected with HIV, although the prevalence of polypharmacy has not been examined thoroughly in older HIV-infected people. One cohort study conducted in 2010 reported that 22% of patients older than 45 years used ≥ 10 drugs, including ART. The greater use of drugs was generally related to a greater need for non-ART medications [8]. In the Veterans Aging Cohort Study, the average number of daily long-term medications increased with age. Among patients 50 years and older, 55% were using ≥ 5 daily medications [9].

The aim of this study was to identify and compare the prevalences of polypharmacy and comorbidities between HIV-infected patients aged 50 years or older and those younger than 50 years in a Mexican population.

## Main text

### Materials and methods

#### Design

We performed a case–control study of adult patients infected with HIV-1. Cases included patients aged 50 years or older who were followed as outpatients for ≥ 6 months and control patients who were treated similarly but were younger than 50 years.

#### Patients

The recruited patients were > 16 years of age, and their infections with HIV-1 had been previously confirmed by enzyme-linked immunosorbent assay (ELISA) and Western blot analysis. The patients were followed as outpatients for at least 6 months and stratified according to age: < 50 years and ≥ 50 years at the time of enrollment in the study.

#### Ethics statement

The study was conducted in accordance with current ethical considerations and was approved for the research protocol from the Ethics and Investigation Committee of “La Raza” National Medical Center (approval number R-2015-3502-148). Written informed consent was required before a patient was given the questionnaire.

#### Measurements

The comorbidities researched included systemic arterial hypertension, dyslipidemia, diabetes mellitus, osteoarthritis, acid peptic disease, chronic kidney disease, heart disease, chronic obstructive pulmonary disease, liver disease, and obesity.

Body mass index (BMI) was defined as body mass divided by the square of body height (kg/m^2^), and obesity was defined as a BMI > 30 kg/m^2^. Osteoarthritis was defined as articular pain lasting > 6 months that was not associated with an uncontrolled or acute disease. Chronic obstructive pulmonary disease was defined by using spirometry and by the presence of post-bronchodilator forced expiratory volume 1 and forced vital capacity (FEV_1_/FVC) < 0.7. Acid peptic disease was defined as symptoms and signs along with consumption of antacids and/or imaging study results that were compatible with the disease, as documented in the clinical file. Liver disease was defined using the set of clinical and laboratory test results for > 6 months that were consistent with the imaging studies that could not be explained by other uncontrolled disease. Chronic kidney disease was defined according to the *Modification in Diet Renal Disease* [10] definition as creatinine clearance < 60 mL/min/1.73 m^2^ for ≥ 6 months with previous ART and without the presence of acute or uncontrolled disease. Dyslipidemia was defined as a serum triglyceride concentration ≥ 150 mg/dL and/or cholesterol concentration ≥ 200 mg/dL in ≥ 2 determinations. Diabetes mellitus was defined as follows: a fasting blood glucose concentration ≥ 126 mg/dL or a blood glucose concentration ≥ 200 mg/dL in any determination, a glycated hemoglobin level ≥ 6.5%, or patient-reported illness and use of antihyperglycemic drugs or insulin. Polypharmacy was defined as the use of five or more concomitant medications. Five concomitant medications is generally accepted as the threshold associated with negative health outcomes [11]. Heart disease was identified as heart failure and/or coronary artery disease. Heart failure was defined based on the symptoms reported by the patient and the prior treatment recorded in the medical history. Coronary artery disease was identified if it was recorded in the clinical record.

#### Statistical analysis

The data were summarized using the median and interquartile range (IQR) for continuous variables and proportion for categorical variables. Bivariate analysis was performed to evaluate the risk for comorbidities and polypharmacy and to obtain the odds ratio (OR) using the Chi-squared test and Fisher’s exact test. Independent risk factors associated with polypharmacy and the number of comorbidities in each group were identified in the logistic regression analysis, which included the significant variables from the bivariate analysis. All analyses were conducted using SPSS software (version 22; SPSS IBM Corp., Armonk, NJ, USA).

### Results

Of the 131 patients invited to participate in the study, 125 agreed to participate, and 60 (48%) of these patients were aged 50 years or older. The median age was 55.5 years (IQR: 52–59.7) in the older group and 31 years (IQR: 26–36) in the younger group. Both groups included fewer women than men: 16% of the older group and 8% of the younger group were women.

The median CD4 + cell counts of patients who were outpatients of the medical center for ≥ 6 months were 509 cells/μL (IQR: 324–730) in the older group and 384 cells/μL (IQR: 262–562) in the younger group (*P* = 0.021); 80% of the older group and 63% of the younger group exhibited viral suppression (*P* = 0.015) (Table 1).

**Table 1:** Profile characteristics of infection (n = 125)

Characteristics	Age < 50 years, n = 65 (IQR*)	Age ≥ 50 years, n = 60 (IQR*)	*P* value
Years since diagnosis	1 (0.8–3.5)	7 (2–16)	< 0.001
CD4+ cell count, cells/mm^3^	384 (262–562)	509 (324–730)	0.021
HIV log_10_ HIV RNA	1.27 (1.27–2.24)	1.27 (1.27–1.27)	0.003
HIV RNA viral load < 50 copies/mL^a^, number (%)	41 (63%)	48 (80%)	0.015
Total number of drugs used, ART plus non-ART	4 (3–5)	6 (4–8)	< 0.001
Total number of ART drugs used^b^	3 (3–4)	4 (3–4)	0.002
Total number of comorbidities found	2 (1–3)	3.5 (2–5)	< 0.001
BMI, kg/m^2^	23.75 (21.61–26.05)	25.52 (22.96–28.51)	0.018
Accumulated exposure/smoking index	0.1 (0–0.75)	0.1 (0–9.7)	< 0.001

*Data are presented as the median (IQR) unless indicated otherwise

^a^Per log_10_ copies/mL increase (centered at 1.30, level of detection)

^b^Only patients with current ART (9 younger patients did not have medically indicated treatment)

The most prevalent drugs used were as follows: antihypertensive agents, which were used by 21 (35%) older patients and 2 (3.1%) younger patients (OR: 11.37; 95% CI 2.75–46.6; *P* < 0.001); hypolipidemic drugs, which were used by 17 (28.3%) older patients and 2 (3.1%) younger patients (OR: 9.2; 95% CI 2.22–38.19; *P* < 0.001); nonsteroidal anti-inflammatory drugs (NSAIDs), which were used by 14 (23.3%) older patients and 4 (6.2%) younger patients (OR: 3.79; 95% CI 1.32–10.88; *P* = 0.006); and anticonvulsants/anxiolytics, which were used by 14 (23.3%) older patients and 4 (6.2%) younger patients (OR: 3.79; 95% CI 1.32–10.88; *P* = 0.009) (Table 2).

**Table 2:** Characteristics of non-ART drugs used (n = 125)

Characteristic	Age < 50 years, n = 65	Age ≥ 50 years, n = 60	Odds ratio (95% CI)	*P* value
Polypharmacy	17 (26.5%)	42 (23.3%)	6.58 (3.01–14.39)	< 0.001
Antihypertensive medication	2 (3.1%)	21 (35%)	11.37 (2.75–46.46)	< 0.001
Antihyperglycemic medication	2 (3.1%)	17 (28.3%)	9.2 (2.22–38.19)	< 0.001
NSAIDs	4 (6.2%)	14 (23.3%)	3.79 (1.32–10.88)	0.009
Anticonvulsants/anxiolytics	4 (6.2%)	14 (23.3%)	3.79 (1.32–10.88)	0.009
Current sildenafil use	6 (9.2%)	14 (23.3%)	2.52 (1.03–6.15)	0.032
Antacid	4 (6.2%)	12 (20%)	3.25 (1.1–9.53)	0.03
Antihyperglycemic agents	1 (1.5%)	11 (18.3%)	11.91 (1.58–89.54)	0.001
Vitamins/minerals	1 (1.5%)	11 (18.3%)	11.91 (1.58–89.54)	0.002
Antidepressants	4 (6.4%)	4 (6.2%)	1.08 (0.28–4.14)	1.0
Antibiotics	11 (16.9%)	3 (5%)	0.29 (0.08–0.98)	0.047
Diuretics	1 (1.5%)	2 (3.3%)	2.16 (0.2–23.28)	0.60
Food supplements	0 (0%)	3 (5%)		0.10

Data are presented as the number (%)

#### Comorbidities

The median number of comorbidities was 2 (IQR: 2–3) in the older group and 1 (IQR: 0–1) in the younger group (*P* ≤ 0.001). The following comorbidities differed significantly between the older and younger groups: systemic arterial hypertension in 20 (33%) of the older patients and 2 (3.1%) of the younger patients (OR: 15.1; 95% CI 3.3–68.1; *P* ≤ 0.001); diabetes mellitus in 11 (18.3%) of the older patients and 1 (1.5%) of the younger patients (OR: 13.8; 95% CI 1.7–110.9; *P* = 0.002); hyperlipidemia in 49 (81.7%) of the older patients and 40 (61.5%) of the younger patients (OR: 2.9; 95% CI 1.2–6.6; *P* = 0.009); osteoarthritis in 20 (33.3%) of the older patients and 3 (4.6%) of the younger patients (OR: 9.9; 95% CI 2.7–35.5; *P* < 0.001); acid peptic disease in 12 (20%) of the older patients and 4 (6.2%) of the younger patients (OR: 3.6; 95% CI 1.1–12.1; *P* = 0.032); and obesity in 11 (18.3%) of the older patients and 3 (4.6%) of the younger patients (OR: 4.4; 95% CI 1.1–16.9; *P* = 0.023) (Table 3).

**Table 3:** Prevalence of the comorbidities investigated (n = 125)

Comorbidity	Age < 50 years, n = 65	Age ≥ 50 years, n = 60	Odds ratio (95% CI)	*P* value
Systemic arterial hypertension	2 (3.1%)	20 (33%)	15.1 (3.3–68.1)	< 0.001
Osteoarthritis	3 (4.6%)	20 (33.3%)	9.9 (2.7–35.5)	< 0.001
Hyperlipidemia	40 (61.5%)	49 (81.7%)	2.9 (1.2–6.6)	0.009
Diabetes mellitus	1 (1.5%)	11 (18.3%)	13.8 (1.7–110.9)	0.002
Acid peptic disease	4 (6.2%)	12 (20%)	3.6 (1.15–12.1)	0.032
Obesity, BMI > 30 kg/m^2^	3 (4.6%)	11 (18.3%)	4.47 (1.18–16.9)	0.023
HBV coinfection	6 (9.2%)	11 (18.3%)	2.12 (0.73–6.16)	0.158
Heart failure and/or coronary heart disease	1 (1.5%)	1 (1.7%)	1.05 (0.64–17.16)	1.0
Chronic kidney disease	3 (4.6%)	4 (6.7%)	1.42 (0.30–6.65)	0.713
Current malignant tumors	2 (2.7%)	4 (6.7%)	2.25 (0.39–12.75)	0.348
Current sexually transmitted disease	21 (32.3%)	11 (18.3%)	0.47 (0.20–1.08)	0.074
Liver disease	0 (0%)	2 (3.3%)		0.138
Chronic obstructive pulmonary disease	0 (0%)	1 (1.7%)		0.296

Data are presented as the number (%)

#### Polypharmacy

Polypharmacy differed significantly between the age groups: 42 (70%) older patients and 17 (26.2%) younger patients met the criteria for polypharmacy (OR: 6.58; 95% CI 3.01–14.39; *P* < 0.001). The median number of drugs used concomitantly was 6 (IQR: 4–8) in the older group and 4 (IQR: 3–5) in the younger group (*P* < 0.001).

After adjustment of the logistic regression model in the older group, the following comorbidities differed between the age groups: systemic arterial hypertension (odds ratio [OR]: 8.8; 95% confidence interval [CI] 1.42–52.72; *P* = 0.017), osteoarthritis (OR: 11.84; 95% CI 2.72–50.9; *P* = 0.001), hyperlipidemia (OR: 2.8; 95% CI 1.03–7.64; *P* = 0.042), and polypharmacy (OR: 6.58; 95% CI 3.01–14.39; *P* = 0.001).

### Discussion

Since 1996, ART has increased the survival rate and number of older people with HIV infection. ART is associated with a decrease in morbidity and mortality related to acquired immunodeficiency syndrome (AIDS) [10]. However, the prevalence of age-related comorbidities and the number of drugs related to the comorbidities have increased unexpectedly in older HIV-infected people [2, 11].

In this study, the prevalence of polypharmacy was significantly higher in the older group than in the younger group. The comorbidities independently associated with older age were systemic arterial hypertension, hyperlipidemia, and osteoarthritis.

According to a prediction, in 2030, 84% of HIV-infected patients will have at least one age-related non-communicable disease and 28% will have three or more [12]. Our data are similar to those of Marzolini et al., who found that older patients were more likely to use one or more comedications than younger patients (82% vs. 61%; *P* < 0.001) [13]. Moreover, as observed in the older group in the current study, the older patients in the study by Marzolini et al. tended to use comedications and certain therapeutic drug classes, such as cardiovascular drugs (53% vs. 19%; *P* < 0.001) and gastrointestinal medications (10% vs. 6%; *P *= 0.004), more often than the younger group. Another study showed that 147 of 165 (89%) patients had comorbid conditions (mean number of conditions, 2.4) and that 133 (81%) received HIV-unrelated medications (mean number of medications, 2.7) [14]. Geriatric syndromes such as cognitive impairment, urinary incontinence, falls, fractures, dementia, and fragility are more likely to occur in the setting of polypharmacy, which is increasingly relevant to HIV care as patients age in the era of combination ART [15, 16].

Hasse et al. reported that comorbidity and multimorbidity associated with non-AIDS diseases, particularly diabetes mellitus, cardiovascular disease, non-AIDS-defining malignancies, and osteoporosis, increase with age and become more important in the care of HIV-infected persons [17].

In this study was shown that HIV-infected individuals aged > 50 years had better virological responses to ART and CD4+ T-cell counts compared with younger patients [18]. These results are consistent with prior studies, who demonstrate better outcomes among older patients [19–21]. While other studies no differences in virological response to ART by age [22–24]; the reason for the discrepancy between the CD4+ cell count and virological responses to ART may be the lag between diagnosis of HIV/AIDS and higher ART adherence was the key factor for older patients, who must overcome potential obstacles to a robust response, including an increased risk of adverse events, a higher comorbidity, and possible age-related immune senescence [10].

A small cross-sectional national health and nutritional examination survey found that HIV-infected elderly patients had higher prevalences of hypertension, hypertriglyceridemia, low bone mineral density, and lipodystrophy than the controls that were matched 1:1 by age, race, gender, smoking status and BMI [24]. This result suggests that the effects of HIV and treatment-related factors contribute to the development of these conditions more than does “normal” aging. Most studies have reported hypertension and cardiovascular risk factors as the main comorbidities; these are also among the leading causes of death worldwide [5, 17, 24].

### Conclusion

Among the comorbidities studied, systemic arterial hypertension, osteoarthritis, hyperlipidemia, and polypharmacy were more frequent in the older patients.

## Limitations

Our study has several limitations. The study included a small sample size, and certain variables could not be controlled. There may have been selection bias in choosing the cases and controls. Another limitation is related to study population, only were included HIV infected patients; therefore, it need more comparative studies with HIV-uninfected people. Most of the patients in both groups were men, hindering the extrapolation of our findings about the prevalence of comorbidities and polypharmacy in women. Moreover, our results can be generalized only to male populations with similar characteristics. However, the data from this study are clinically relevant given the lack of similar studies on the prevalence of comorbidities and polypharmacy in the older Mexican HIV-infected population. Observational studies may more closely approximate the prevalence of specific diseases in clinical practice. The case–control design allowed us to estimate the relative risks for diseases or events, which was the aim of our study. Further prospective studies of elderly patients are needed to individualize treatment for patients with polypharmacy and comorbidities.

## Data Availability

All data generated or analyzed during this study are included in this published article and its additional information files.

## Abbreviations

AIDS: acquired immuno deficiency syndrome
ART: antiretroviral therapy
BMI: body mass index
CI: confidence interval
ELISA: enzyme-linked immunosorbent assay
FEV_1_: forced expiratory volume 1
FVC: forced vital capacity
HIV-1: human immunodeficiency virus
IQR: interquartile range
NSAIDs: nonsteroidal anti-inflammatory drug
OR: odds ratio
